# Effectiveness and safety of four different beta‐blockers in patients with chronic heart failure

**DOI:** 10.1002/mco2.199

**Published:** 2023-01-06

**Authors:** Baoshan Liu, Rui Zhang, Aiyuan Zhang, Guodong Wang, Jiupan Xu, Yun Zhang, Yanping Liu, Panpan Hao

**Affiliations:** ^1^ Department of Cardiology Qilu Hospital of Shandong University in Jinan, and Department of Cardiology Weifang People's Hospital Weifang Shandong P. R. China; ^2^ Department of Cardiology Key Laboratory of Cardiovascular Remodeling and Function Research Chinese Ministry of Education Chinese National Health Commission and Chinese Academy of Medical Sciences State and Shandong Province Joint Key Laboratory of Translational Cardiovascular Medicine Qilu Hospital of Shandong University Jinan Shandong P. R. China; ^3^ Department of Radiology Qilu Hospital of Shandong University Jinan Shandong P. R. China

## Abstract

In this study, we evaluated the effectiveness and safety of bisoprolol, metoprolol, carvedilol, and nebivolol in the treatment of chronic heart failure. The results demonstrated that bisoprolol improved the prognosis of chronic heart failure in comparison with carvedilol, and carvedilol exerted similar effects as metoprolol succinate and nebivolol and better effect than metoprolol tartrate (evidence levels: bisoprolol > carvedilol = metoprolol succinate = nebivolol > metoprolol tartrate; “ > ” means “prior to”).

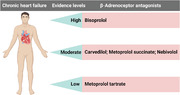

Dear Editor,

Initiation with a very low dose of β blockers and titration to the tolerated dose is recommended as the cornerstone in the treatment of chronic heart failure (CHF) with reduced ejection fraction, but which β blocker has the superiority over others remains unclear.^1^ It is necessary to perform an all‐sided pooled analysis that can exploit the totality of evidence stemming from randomized controlled trials (RCTs) and observational studies to compare the efficacy and safety of different β blockers and determine an optimal selection in the treatment of CHF.

Twenty‐six studies were deemed eligible for inclusion, including 14 RCTs and 12 observational studies (Figure S1). The methodological quality of these included studies was good generally (Table S1).

Compared with carvedilol, bisoprolol decreased all‐cause mortality (odds ratio [OR], 1.25; 95% confidence interval [CI], 1.11–1.42; *p* = 0.0002) (Figure 1A), but did not have a significant decrease in heart failure‐related readmission in CHF patients (OR, 1.95; 95% CI, 0.65–5.82; *p* = 0.23) (Table S2). Compared with carvedilol, bisoprolol was associated with a significant increase in left ventricular ejection fraction (LVEF) (% of change; pooled weighted mean difference [WMD]; –4.98, 95% CI, –7.11––2.84; *p* < 0.00001). However, bisoprolol and carvedilol had similar effects in decreasing heart rate (% of change; WMD; −5.94, 95% CI, −13.92–2.05; *p* = 0.15) and hospital stay (days; WMD; 1.98, 95% CI, −0.26–4.22; *p* = 0.08). No differences were observed in total drug−related adverse events (OR, 0.97; 95% CI, 0.76 to 1.23; *p* = 0.78), including worsening heart failure (OR, 0.91; 95% CI, 0.67–1.22; *p* = 0.52) and hypotension (OR, 1.16; 95% CI, 0.74–1.82; *p* = 0.51), but carvedilol caused fewer bradycardia (OR, 0.60; 95% CI, 0.41–0.89; *p* = 0.01) in comparison with bisoprolol (Table S2). Compared with carvedilol, bisoprolol decreased all−cause mortality in Asian patients, with an OR of 1.28 (95% CI, 1.03–1.58; *p* = 0.02), but not in Caucasian patients (OR 0.95; 95% CI, 0.55–1.63; *p* = 0.85); bisoprolol decreased all−cause mortality in patients with mean LVEF < 40% (OR 1.32; 95% CI, 1.15–1.53; *p* = 0.0001), but not in those with LVEF ≥ 40% (OR 0.93; 95% CI, 0.52–1.66; *p* = 0.69) (Table S2). Thus, bisoprolol has superior benefit and similar safety for CHF in comparison with carvedilol.

**FIGURE 1:**
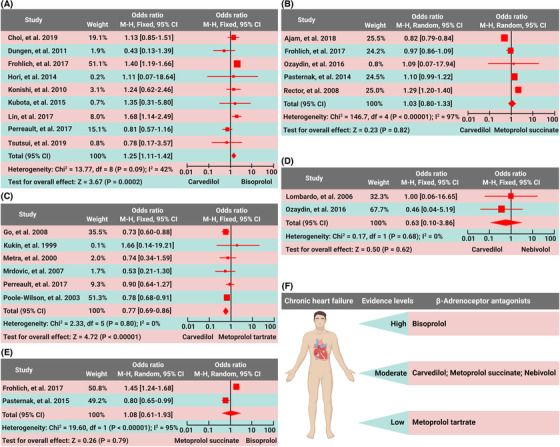
Data summaries from the trials of different β blockers for chronic heart failure (CHF). (A) Compared with carvedilol, bisoprolol decreased all−cause mortality (odds ratio [OR], 1.25; 95% confidence interval [CI], 1.11–1.42; *p* = 0.0002) in CHF patients. (B) There was no difference in all−cause mortality (OR, 1.03; 95% CI, 0.80–1.33; *p* = 0.82) between the carvedilol group and the metoprolol succinate group in CHF patients. (C) Compared with metoprolol tartrate, carvedilol decreased all−cause mortality (OR, 0.77; 95% CI, 0.69–0.86; *p* < 0.00001) in CHF patients. (D) There was no difference in all−cause mortality (OR, 0.63; 95% CI, 0.10–3.86; *p* = 0.62) between the carvedilol group and the nebivolol group in CHF patients. (E) Compared with metoprolol succinate, bisoprolol did not significantly decrease all−cause mortality (OR, 1.08; 95% CI, 0.61–1.93; *p* = 0.79) in CHF patients. (F) Evidence levels of different β blockers in the treatment of CHF

There were no differences in all‐cause mortality (OR, 1.03; 95% CI, 0.80–1.33; *p* = 0.82), repeat hospitalization (per 100 patient−years) (WMD, 2.50; 95% CI, −3.02–8.02; *p* = 0.38) or LVEF (WMD, −0.02; 95% CI, −0.32–0.29; *p* = 0.91) between the carvedilol group and the metoprolol succinate group (Figure 1B and Table S3). Thus, metoprolol succinate and carvedilol have similar evidence in the treatment of CHF.

Compared with metoprolol tartrate, carvedilol decreased all‐cause mortality (OR, 0.77; 95% CI, 0.69–0.86; *p* < 0.00001) but not heart failure readmission in CHF patients (OR, 1.0; 95% CI, 0.87–1.15; *p* = 0.99) (Figure 1C). Compared with metoprolol tartrate, carvedilol increased LVEF (WMD, 4.83; 95% CI, 2.43–7.22; *p* < 0.0001), but did not affect 6‐min‐walk distance (6MWD) (WMD, −12.47, 95% CI, −60.28–35.34; *p* = 0.61), heart rate (WMD, −1.59; 95% CI, −7.41–4.24; *p* = 0.59), or quality of life assessed by the Minnesota Living with Heart Failure Questionnaire score (WMD, −5.57; 95% CI, −11.4–0.26; *p* = 0.06). Carvedilol reduced worsening heart failure in comparison with metoprolol tartrate (OR, 0.41; 95% CI, 0.18–0.98; *p* = 0.04), and no differences were observed in the incidences of total side effects and drug withdrawals between the two groups (OR, 0.91; 95% CI, 0.72–1.16; *p* = 0.45) (Table S4). Thus, carvedilol provides more benefit than metoprolol tartrate in the treatment of CHF, with a similar safety profile.

There were no differences in all‐cause mortality (OR, 0.63; 95% CI, 0.10–3.86; *p* = 0.62), hospital readmission due to heart failure (OR, 1.31; 95% CI, 0.52–3.28; *p* = 0.57) or drug‐related adverse events (OR, 1.09; 95% CI, 0.53–2.27; *p* = 0.81) between the carvedilol group and the nebivolol group (Figure 1D and Table S5). Thus, similar to carvedilol, nebivolol is a faith‐worthy β blocker in CHF patients.

Compared with metoprolol succinate, bisoprolol did not significantly decrease all‐cause mortality (OR, 1.08; 95% CI, 0.61–1.93; *p* = 0.79) in CHF patients (Figure 1E), though the readmission rate was lower in bisoprolol users than metoprolol users (OR, 1.29; 95% CI, 1.03–1.63; *p* = 0.03) (Table S6).

Although 50%–60% of adrenergic signaling pathways are inactivated in patients with advanced heart failure, numerous receptors are still exposed to excessive catecholamines, resulting in cardiac remodeling and dysfunction. β1 receptor activation causes intracellular cAMP‐mediated Ca^2+^ overload and increases myocardial oxygen consumption and damage. Compared to the β1 receptor, α receptor‐associated cardiac remodeling is weaker due to its lower density in the heart. However, after α receptor activation, vascular smooth muscle contracts, peripheral vascular resistance and cardiac afterload increase, and heart failure progression are accelerated.^2^

Bisoprolol, metoprolol, and nebivolol are selective β1 blockers with affinity ratios of β1 to β2 about 103, 74, and 321, respectively, and carvedilol acts as a non‐selective α1 and β blocker with β1/β2 selectivity almost 1:1.^3^ Bisoprolol has a long elimination half‐life (about 11 h in the healthy population and 17 h in CHF patients, respectively) and no intrinsic sympathomimetic activity.^3^ Metoprolol was found to have an elimination half‐life of 3–4 h and exert the maximum β1 receptor blockade in the blood concentration range of 300–400 nmol/L, beyond which the β2 receptor would be blocked, resulting in adverse reactions. Metoprolol succinate tablet has greater liposolubility than tartrate tablet. In a short period after administration of metoprolol tartrate, the rapid absorption and high bioavailability can cause a peak blood concentration beyond the best efficacy range, resulting in β2 receptor blockade. However, at the end of the interval, blood concentration cannot reach the therapeutic level, leading to insufficient β1 blockade. Compared with metoprolol tartrate, metoprolol succinate shows a more stable β1 blockade effect.^3^ Both carvedilol and nebivolol are third‐generation β blockers with an elimination half‐life of 7–10 and 12–19 h, respectively.^3^, ^4^ Bisoprolol, metoprolol succinate and nebivolol can be administered once daily due to long half‐life or drug release, which greatly improves drug compliance. Although carvedilol needs to be administered twice daily, some studies have shown that carvedilol does outperform metoprolol in treating CHF, which might be attributed to additional benefit from its α1 receptor blocking.^5^ There is a lack of scientific evidence that the beneficial effects of the above drugs are extensible to other β blockers, such as propranolol, which has lower receptor selectivity and shorter elimination half‐life.

In summary, carvedilol exerts similar benefits as metoprolol succinate and nebivolol in CHF patients. Bisoprolol is considerably more effective than the other several β blockers, and the curative effect of metoprolol tartrate is worse than the above several β blockers (Figure 1F). There are similar incidences of adverse reactions among different β blockers. However, head‐to‐head comparative trials on the efficacy and safety of different β blockers in CHF patients are awaited.

## AUTHOR CONTRIBUTIONS

BL, YZ and PH conceived the protocol and drafted the manuscript. BL, RZ, JX, YL, and PH collected the literatures, extracted and analyzed the data, and substantively revised the manuscript drafts. Data curation was performed by BL, RZ, AZ, GW, JX, and YL. All authors contributed to the final revision of manuscript drafts and approved this manuscript for publication.

## CONFLICT OF INTEREST

The authors declare that they have no conflict of interest.

## FUNDING INFORMATION

This work was supported by the Natural Science Foundation of Shandong Province (ZR2021QH262, ZR2020QH005, ZR2019QH001), the Natural Science Foundation of Jiangsu Province (BK20200224).

### ETHICS APPROVAL

Not applicable.

## Supporting information



Supporting Information

## Data Availability

Not applicable.
