# Supplementary material for: Effectiveness and safety of four different beta‐blockers in patients with chronic heart failure
Source: MedComm (2020). 2023 Jan 6;4(1):e199. doi: 10.1002/mco2.199 (PMC9823244; doi:10.1002/mco2.199)
Supplement: Supplementary file 1 — Supporting Information [file MCO2-4-e199-s001.docx]

**Supporting Information for**

**Effectiveness and safety of 4 different beta-blockers in patients with chronic heart failure**

Baoshan Liu^1#^, Rui Zhang^1#^, Aiyuan Zhang^1^, Guodong Wang^1^, Jiupan Xu^2^, Yun Zhang^2^, Yanping Liu^3^*, Panpan Hao^2^*

**From:**

^1^Department of Cardiology, Qilu Hospital of Shandong University & Department of Cardiology, Weifang People's Hospital, Weifang, Shandong, P. R. China

^2^Department of Cardiology, Key Laboratory of Cardiovascular Remodeling and Function Research, Chinese Ministry of Education, Chinese National Health Commission and Chinese Academy of Medical Sciences, State & Shandong Province Joint Key Laboratory of Translational Cardiovascular Medicine, Qilu Hospital of Shandong University, Jinan, Shandong, P. R. China

^3^Department of Radiology, Qilu Hospital of Shandong University, Jinan, Shandong, P. R. China

* **Corresponding Authors:**

Panpan Hao − Department of Cardiology, Key Laboratory of Cardiovascular Remodeling and Function Research, Chinese Ministry of Education, Chinese National Health Commission and Chinese Academy of Medical Sciences, State & Shandong Province Joint Key Laboratory of Translational Cardiovascular Medicine, Qilu Hospital of Shandong University, Jinan 250012, Shandong, P. R. China.

E‑mail: [panda.how@sdu.edu.cn](mailto:panda.how@sdu.edu.cn)

Yanping Liu − Department of Radiology, Qilu Hospital of Shandong University, Jinan 250012, Shandong, P. R. China.

E‑mail: [llliuyanping@126.com](mailto:llliuyanping@126.com)

^#^ These authors contributed equally to this work.

**Methods and Materials**

**Study search and selection**

We systematically searched the following electronic databases: MEDLINE, Embase, J-STAGE, Springer, the Cochrane Library, and the grey literature (SIGLE) database. Also, we searched for studies on these websites: http://www.clinicaltrials.gov, http://www.clinicaltrialresults.org, <http://www.cardiosource.org/acc>, http://www.americaheart.org, and http://www.escardio.org. We did not restrict studies on language or whether the results had been published. Searches were updated using automated e-mail alerts until December 2021.

The search algorithm for MEDLINE was as follows: (“heart failure” or “cardiac dysfunction”) and (“adrenergic beta antagonists” or “β blocker” or “carvedilol” or “metoprolol” or “bisoprolol” or “nebivolol”). Similar but adapted search terms were used for other databases or search engines.

Studies were selected for inclusion based on the following criteria: (1) study design: randomized controlled trials or observational studies; (2) study population: adult CHF patients; (3) at least two common β blockers were compared, including carvedilol, metoprolol succinate, metoprolol tartrate, bisoprolol, and nebivolol; (4) outcomes: death, readmission due to heart failure, heart rate, left ventricular ejection fraction (LVEF), 6-min-walk distance (6MWD), the Minnesota Living with Heart Failure Questionnaire (MLHFQ), the 36-Item Short Form Health Survey (SF-36), drug-related adverse events, and so on. Studies were excluded based on the following criteria: (1) two articles reported results of the same study (the article with more complete and recent data was included); (2) β blockers were not classified clearly; (3) follow-up in either group was less than 4 weeks. Two authors (Baoshan Liu and Rui Zhang) searched for and reviewed articles independently, and those studies that met the inclusion criteria were selected for further analyses.

**Assessment of study quality**

The methodological quality of eligible studies was assessed with the criteria we provided in a previous study. The criteria were as follows: enrollment of unselected subjects; a clearly defined inception cohort (i.e. patients enrolled at the time of the initial diagnosis or at a uniform time after diagnosis); more than 90% of patients completed planned follow-ups; and blinded outcome assessment.

The literature search, data extraction, and quality assessment were undertaken independently and blindly by two authors (Baoshan Liu and Rui Zhang) using a standardized approach. Any disagreements were resolved by a third reviewer (Panpan Hao).

**Statistical analysis**

RevMan 5.3 software was used for the pooled analyses. The heterogeneity between studies was tested by the chi-square-based Cochran’s Q statistic and the inconsistency index (*I^2^*). Statistically, significant heterogeneity was considered present with a chi-square *P* < 0.05 and *I^2^* > 50%. Results showing no significant heterogeneity were analyzed by the fixed-effects model and those with significant heterogeneity were analyzed by the random-effects model. To analyze the origin of heterogeneity and decrease heterogeneity, subgroup analyses were conducted to seek more narrowly drawn subsets of reports of studies with similar features to assess individual effects, according to study design, race, LVEF, and the dosage of β blocker. Pooled weighted mean difference (WMD) or odds ratio (OR) was reported with its corresponding 95% confidence interval (CI), and a two-tailed *P* < 0.05 was considered statistically significant.


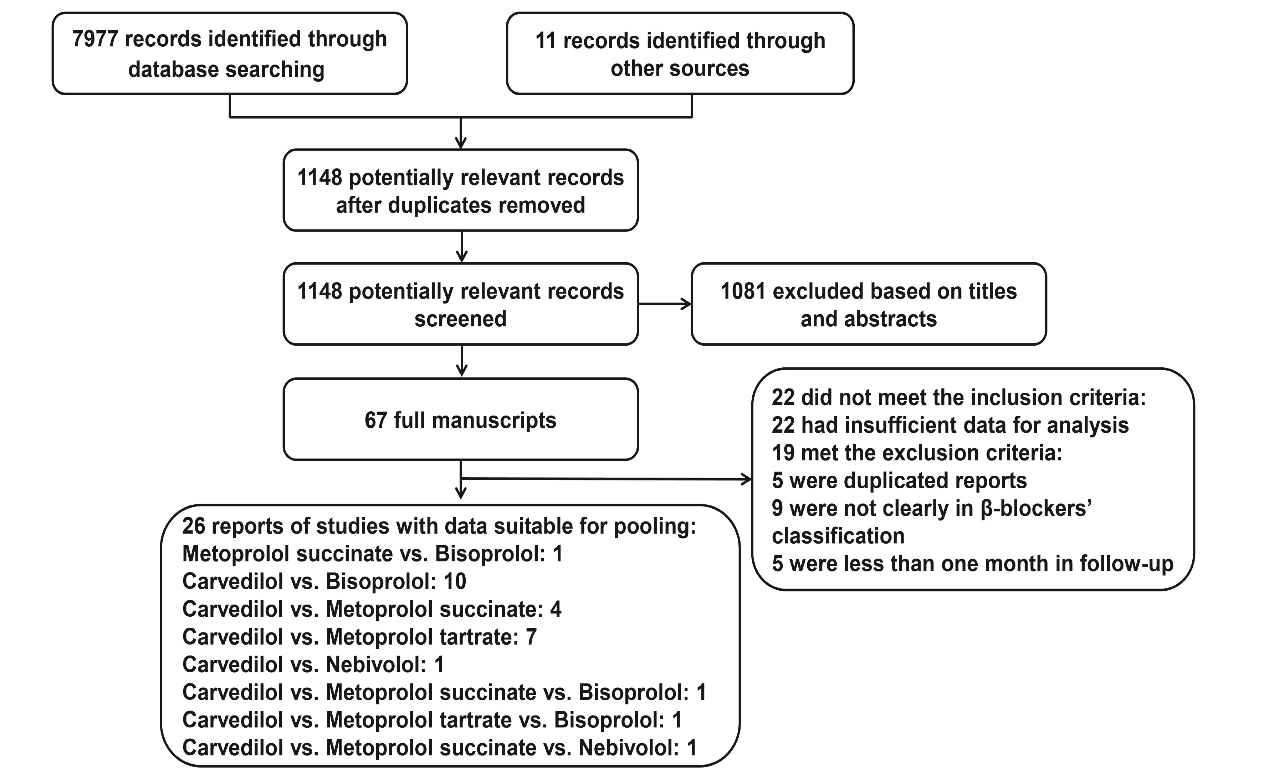


**Figure S1. Flowchart of literature selection process.**

**Table S1. Assessment of study quality.**

| β-blocker comparison | Study | Study type | Consecutive patients | Clearly defined inception cohort | > 90% completed planned follow-up (%) | Blinded outcome assessment |
| --- | --- | --- | --- | --- | --- | --- |
| Carvedilol vs. bisoprolol | Aygul, et al. 2009^1^ | RCT | Yes | Yes | 100.0 | Yes |
|  | Konishi, et al. 2010^15^ | Observational study | Yes | Yes | 100.0 | No |
|  | Dungen, et al. 2011^2^ | RCT | Yes | Yes | 89.2 | Yes |
|  | Lainscak, et al. 2011^3^ | RCT | Unclear | Yes | 87.0 | No |
|  | Marazzi, et al. 2011^4^ | RCT | Yes | Yes | 93.1 | Yes |
|  | Hori, et al. 2014^5^ | RCT | Yes | Yes | 59.3 | Yes |
|  | Kubota, et al. 2015^16^ | Observational study | Yes | Yes | 100.0 | No |
|  | Perreault, et al. 2017^17^ | Observational study | Yes | Yes | 100.0 | No |
|  | Frohlich, et al. 2017^18^ | Observational study | Yes | Yes | 100.0 | No |
|  | Choi, et al. 2019^19^ | Observational study | Yes | Yes | 100.0 | No |
|  | Tsutsui, et al. 2019^6^ | RCT | Yes | Yes | 92.6 | No |
|  | Lin, et al. 2017^20^ | Observational study | Yes | Yes | 100.0 | No |
| Carvedilol vs. metoprolol succinate | Ajam, et al. 2018^21^ | Observational study | Yes | Yes | 100.0 | No |
|  | Fröhlich, et al. 2017^18^ | Observational study | Yes | Yes | 100.0 | No |
|  | Ozaydin, et al. 2016^7^ | RCT | Yes | Yes | 91.9 | Yes |
|  | Pasternak, et al. 2014^22^ | Observational study | Yes | Yes | 100.0 | No |
|  | Shore, et al. 2012^23^ | Observational study | Yes | Yes | 100.0 | No |
|  | Rector, et al. 2008^24^ | Observational study | Yes | Yes | 100.0 | No |
| Carvedilol vs. metoprolol tartrate | Kukin, et al. 1999^8^ | RCT | Yes | Yes | 89.6 | Yes |
|  | Metra, et al. 2000^9^ | RCT | Unclear | Yes | 81.3 | Yes |
|  | Poole-Wilson, et al. 2003^10^ | RCT | Yes | Yes | 100.0 | Yes |
|  | Mrdovic, et al. 2007^11^ | RCT | Yes | Yes | 93.0 | No |
|  | Go, et al. 2008^25^ | Observational study | Yes | Yes | 100.0 | No |
|  | Shahzamani, et al. 2011^12^ | RCT | Yes | Yes | 100.0 | Yes |
|  | Perreault, et al. 2017^17^ | Observational study | Yes | Yes | 100.0 | No |
|  | Sanderson, et al. 2005^13^ | RCT | Yes | Yes | 100.0 | Yes |
| Carvedilol vs. nebivolol | Lombardo, et al. 2006^14^ | RCT | Unclear | Yes | 100.0 | Yes |
|  | Ozaydin, et al. 2016^7^ | RCT | Yes | Yes | 91.9 | Yes |
| Metoprolol succinate vs. bisoprolol | Pasternak, et al. 2015^26^ | Observational study | Yes | Yes | 100.0 | No |
|  | Frohlich, et al. 2017^18^ | Observational study | Yes | Yes | 100.0 | No |

**Table S2. Carvedilol versus bisoprolol in chronic heart failure.**

| Outcomes | Patients (n) | OR/WMD (95% CI) | *P* value | I^2^ (%) | Heterogeneity *P* value |
| --- | --- | --- | --- | --- | --- |
| All-cause mortality | 8227 | 1.25 [1.11, 1.42] | 0.0002 | 42 | 0.09 |
| Subgroup analyses | | | | | |
| Asians | 3279 | 1.28 [1.03, 1.58] | 0.02 | 0 | 0.70 |
| Caucasians | 4948 | 0.95 [0.55, 1.63] | 0.85 | 81 | 0.005 |
| Mean LVEF ≥40% | 1093 | 0.93 [0.52, 1.66] | 0.69 | 57 | 0.13 |
| Mean LVEF <40% | 4063 | 1.32 [1.15, 1.53] | 0.0001 | 0 | 0.56 |
| HFrEF | 263 | 1.12 [0.85, 1.48] | 0.42 | 0 | 0.89 |
| HF readmission | 4164 | 1.95 [0.65, 5.82] | 0.23 | 89 | <0.00001 |
| Subgroup analyses | | | | | |
| RCT | 1152 | 1.00 [0.56, 1.81] | 0.99 | 0 | 0.57 |
| Observational study | 3012 | 4.82 [0.91, 25.57] | 0.06 | 94 | <0.0001 |
| HFrEF | 276 | 0.95 [0.38, 2.37] | 0.91 | 13 | 0.28 |
| LVEF (% of change) | 298 | -4.98 [-7.11, -2.84] | <0.00001 | 0 | 0.91 |
| Heart rate (% of change) | 298 | -5.94 [-13.92, 2.05] | 0.15 | 88 | 0.004 |
| Hospital stay (days) | 1314 | 1.98 [-0.26, 4.22] | 0.08 | 78 | 0.03 |
| Drug-related adverse events | 1215 | 0.97 [0.76, 1.23] | 0.78 | 37 | 0.19 |
| Subgroup analyses | | | | | |
| Asians | 276 | 0.82 [0.49, 1.37] | 0.45 | 0 | 0.63 |
| Caucasians | 939 | 1.50 [0.48, 4.74] | 0.49 | 75 | 0.04 |
| HFrEF | 122 | 2.01 [0.86, 4,69] | 0.11 | 27 | 0.24 |
| Worsening HF | 1152 | 0.91 [0.67, 1.22] | 0.52 | 0 | 0.42 |
| Bradycardia | 939 | 0.60 [0.41, 0.89] | 0.01 | 0 | 0.72 |
| Hypotension | 939 | 1.16 [0.74, 1.82] | 0.51 | 0 | 0.91 |

**Table S3. Carvedilol versus metoprolol succinate in chronic heart failure.**

| Outcomes | Patients (n) | OR/WMD (95% CI) | *P* value | I^2^ (%) | Heterogeneity *P* value |
| --- | --- | --- | --- | --- | --- |
| All-cause mortality | 130758 | 1.03 [0.80, 1.33] | 0.82 | 97 | <0.00001 |
| HFrEF subgroup | 104533 | 0.95 [0.78, 1.16] | 0.62 | 94 | <0.00001 |
| Readmission (per 100 patient-years) | 3550 | 2.50 [-3.02, 8.02] | 0.38 | 0 | 0.86 |
| LVEF (%) | 3550 | -0.02 [-0.32, 0.29] | 0.91 | 39 | 0.20 |

**Table S4. Carvedilol versus metoprolol tartrate in chronic heart failure.**

| Outcomes | Patients (n) | OR/WMD (95% CI) | *P* value | I^2^ (%) | Heterogeneity *P* value |  |
| --- | --- | --- | --- | --- | --- | --- |
| All-cause mortality | 10049 | 0.77 [0.69, 0.86] | <0.00001 | 0 | 0.80 |  |
| Subgroup analyses | | | | | | |
| RCT | 3509 | 0.78 [0.67, 0.90] | 0.0005 | 0 | 0.77 |  |
| Observational study | 6540 | 0.77 [0.65, 0.90] | 0.001 | 17 | 0.27 |  |
| HFrEF | 3218 | 0.78 [0.68, 0.91] | 0.001 | 0 | 0.83 |  |
| HF readmission | 5613 | 1.00 [0.87, 1.15] | 0.99 | 46 | 0.13 |  |
| Subgroup analyses | | | | | | |
| RCT | 3442 | 0.96 [0.83,1.11] | 0.58 | 40 | 0.19 |  |
| HFrEF | 3442 | 0.96 [0.83,1.11] | 0.58 | 40 | 0.19 |  |
| LVEF (%) | 300 | 4.83 [2.43, 7.22] | <0.0001 | 56 | 0.08 |  |
| Subgroup analyses | | | | | | |
| Caucasians | 249 | 3.95 [1.85, 6.06] | 0.0002 | 20 | 0.29 |  |
| HFrEF | 137 | 4.21 [0.77, 7.64] | 0.0003 | 43 | 0.19 |  |
| 6MWD (m) | 173 | -12.47 [-60.28, 35.34] | 0.61 | 76 | 0.04 |  |
| Heart rate (bpm) | 118 | -1.59 [-7.41, 4.24] | 0.59 | 83 | 0.01 |  |
| MLHFQ score | 189 | -5.57 [-11.40, 0.26] | 0.06 | 45 | 0.18 |  |
| Drug-related adverse events | 3479 | 0.91 [0.72, 1.16] | 0.45 | 0 | 0.78 |  |
| Subgroup analyses | | | | | | |
| Metoprolol tartrate >100mg | 383 | 0.79 [0.52, 1.20] | 0.26 | 0 | 0.64 |  |
| Metoprolol tartrate ≤100mg | 3096 | 0.98 [0.73, 1.31] | 0.89 | 0 | 0.67 |  |
| HFrEF | 3218 | 0.97 [0.74, 1.28] | 0.84 | 0 | 0.91 |  |
| Worsening HF | 189 | 0.41 [0.18, 0.98] | 0.04 | 0 | 0.92 |  |
| Hypotension | 3151 | 1.40 [1.13, 1.74] | 0.002 | 0 | 0.74 |  |
| Bradycardia | 3151 | 1.09 [0.85, 1.39] | 0.51 | 0 | 0.71 |  |

**Table S5. Carvedilol versus nebivolol in chronic heart failure.**

| Outcomes | Patients (n) | OR (95% CI) | *P* value | I^2^ (%) | Heterogeneity *P* value |
| --- | --- | --- | --- | --- | --- |
| All-cause mortality | 184 | 0.63 [0.10, 3.86] | 0.62 | 0 | 0.68 |
| HF readmission | 184 | 1.31 [0.52, 3.28] | 0.57 | 0 | 0.52 |
| Drug-related adverse events | 184 | 1.09 [0.53, 2.27] | 0.81 | 0 | 0.34 |

**Table S6. Metoprolol succinate versus bisoprolol in chronic heart failure.**

| Outcomes | Patients (n) | OR (95% CI) | *P* value | I^2^ (%) | Heterogeneity *P* value |
| --- | --- | --- | --- | --- | --- |
| All-cause mortality | 10638 | 1.08 [0.61, 1.93] | 0.79 | 95 | <0.00001 |
| HF readmission | 6349 | 1.29 [1.03, 1.63] | 0.03 | NA | NA |

**RCTs included in this pooled analysis:**

1. Aygul N, Ozdemir K, Duzenli MA, Aygul MU. The comparative effects of long-term carvedilol versus bisoprolol therapy on QT dispersion in patients with chronic heart failure. *Cardiology* 2009;**112**:168-173. doi: 10.1159/000147950

2. Düngen HD, Apostolovic S, Inkrot S*, et al.* Titration to target dose of bisoprolol vs. carvedilol in elderly patients with heart failure: the CIBIS-ELD trial. *Eur J Heart Fail* 2011;**13**:670-680. doi: 10.1093/eurjhf/hfr020

3. Lainscak M, Podbregar M, Kovacic D, Rozman J, von Haehling S. Differences between bisoprolol and carvedilol in patients with chronic heart failure and chronic obstructive pulmonary disease: a randomized trial. *Respir Med* 2011;**105 Suppl 1**:S44-49. doi: 10.1016/s0954-6111(11)70010-5

4. Marazzi G, Iellamo F, Volterrani M*, et al.* Comparison of effectiveness of carvedilol versus bisoprolol for prevention of postdischarge atrial fibrillation after coronary artery bypass grafting in patients with heart failure. *Am J Cardiol* 2011;**107**:215-219. doi: 10.1016/j.amjcard.2010.08.062

5. Hori M, Nagai R, Izumi T, Matsuzaki M. Efficacy and safety of bisoprolol fumarate compared with carvedilol in Japanese patients with chronic heart failure: results of the randomized, controlled, double-blind, Multistep Administration of bisoprolol IN Chronic Heart Failure II (MAIN-CHF II) study. *Heart Vessels* 2014;**29**:238-247. doi: 10.1007/s00380-013-0340-3

6. Tsutsui H, Momomura SI, Masuyama T*, et al.* Tolerability, Efficacy, and Safety of Bisoprolol vs. Carvedilol in Japanese Patients With Heart Failure and Reduced Ejection Fraction　- The CIBIS-J Trial. *Circ J* 2019;**83**:1269-1277. doi: 10.1253/circj.CJ-18-1199

7. Ozaydin M, Yucel H, Kocyigit S*, et al.* Nebivolol versus Carvedilol or Metoprolol in Patients Presenting with Acute Myocardial Infarction Complicated by Left Ventricular Dysfunction. *Med Princ Pract* 2016;**25**:316-322. doi: 10.1159/000446184

8. Kukin ML, Kalman J, Charney RH*, et al.* Prospective, randomized comparison of effect of long-term treatment with metoprolol or carvedilol on symptoms, exercise, ejection fraction, and oxidative stress in heart failure. *Circulation* 1999;**99**:2645-2651. doi: 10.1161/01.cir.99.20.2645

9. Metra M, Giubbini R, Nodari S*, et al.* Differential effects of beta-blockers in patients with heart failure: A prospective, randomized, double-blind comparison of the long-term effects of metoprolol versus carvedilol. *Circulation* 2000;**102**:546-551. doi: 10.1161/01.cir.102.5.546

10. Poole-Wilson PA, Swedberg K, Cleland JG*, et al.* Comparison of carvedilol and metoprolol on clinical outcomes in patients with chronic heart failure in the Carvedilol Or Metoprolol European Trial (COMET): randomised controlled trial. *Lancet* 2003;**362**:7-13. doi: 10.1016/s0140-6736(03)13800-7

11. Mrdovic IB, Savic LZ, Perunicic JP*, et al.* Randomized active-controlled study comparing effects of treatment with carvedilol versus metoprolol in patients with left ventricular dysfunction after acute myocardial infarction. *Am Heart J* 2007;**154**:116-122. doi: 10.1016/j.ahj.2007.03.049

12. Shahzamani M, Ghanavati A, Froutagheh AN*, et al.* Carvedilol compared with metoprolol on left ventricular ejection fraction after coronary artery bypass graft. *J Perianesth Nurs* 2011;**26**:384-387. doi: 10.1016/j.jopan.2011.09.005

13. Sanderson JE, Leung LY, Chan SK*, et al.* Do metoprolol and carvedilol have equivalent effects on diurnal heart rate in patients with chronic heart failure? *Eur J Heart Fail* 2005;**7**:874-877. doi: 10.1016/j.ejheart.2005.03.002

14. Lombardo RM, Reina C, Abrignani MG*, et al.* Effects of nebivolol versus carvedilol on left ventricular function in patients with chronic heart failure and reduced left ventricular systolic function. *Am J Cardiovasc Drugs* 2006;**6**:259-263. doi: 10.2165/00129784-200606040-00006

**Observational studies included in this pooled analysis:**

15. Konishi M, Haraguchi G, Kimura S*, et al.* Comparative effects of carvedilol vs bisoprolol for severe congestive heart failure. *Circ J* 2010;**74**:1127-1134. doi: 10.1253/circj.cj-09-0989

16. Kubota Y, Asai K, Furuse E*, et al.* Impact of β-blocker selectivity on long-term outcomes in congestive heart failure patients with chronic obstructive pulmonary disease. *Int J Chron Obstruct* Pulmon Dis 2015;10:515-523. doi: 10.2147/copd.s79942

17. Perreault S, de Denus S, White M*, et al.* Older adults with heart failure treated with carvedilol, bisoprolol, or metoprolol tartrate: risk of mortality. *Pharmacoepidemiol Drug Saf* 2017;**26**:81-90. doi: 10.1002/pds.4132

18. Fröhlich H, Torres L, Täger T*, et al.* Bisoprolol compared with carvedilol and metoprolol succinate in the treatment of patients with chronic heart failure. *Clin Res Cardiol* 2017;**106**:711-721. doi: 10.1007/s00392-017-1115-0

19. Choi KH, Lee GY, Choi JO*, et al.* The mortality benefit of carvedilol versus bisoprolol in patients with heart failure with reduced ejection fraction. *Korean J Intern Med* 2019;**34**:1030-1039. doi: 10.3904/kjim.2018.009

20. Lin TY, Chen CY, Huang YB. Evaluating the effectiveness of different beta-adrenoceptor blockers in heart failure patients. Int J Cardiol 2017;230:378-383. doi: 10.1016/j.ijcard.2016.12.098

21. Ajam T, Ajam S, Devaraj S, et al. Effect of carvedilol vs metoprolol succinate on mortality in heart failure with reduced ejection fraction. Am Heart J. 2018;199:1-6.

22. Pasternak B, Svanström H, Melbye M, Hviid A. Association of treatment with carvedilol vs metoprolol succinate and mortality in patients with heart failure. *JAMA Intern Med* 2014;**174**:1597-1604. doi: 10.1001/jamainternmed.2014.3258

23. Shore S, Aggarwal V, Zolty R. Carvedilol or sustained-release metoprolol for congestive heart failure: a comparative effectiveness analysis. *J Card Fail* 2012;**18**:919-924. doi: 10.1016/j.cardfail.2012.10.016

24. Rector TS, Anand IS, Nelson DB, Ensrud KE. Carvedilol versus controlled-release metoprolol for elderly veterans with heart failure. *J Am Geriatr Soc* 2008;**56**:1021-1027. doi: 10.1111/j.1532-5415.2008.01682.x

25. Go AS, Yang J, Gurwitz JH*, et al.* Comparative effectiveness of different beta-adrenergic antagonists on mortality among adults with heart failure in clinical practice. *Arch Intern Med* 2008;**168**:2415-2421. doi: 10.1001/archinternmed.2008.506

26. Pasternak B, Mattsson A, Svanström H, Hviid A. Comparative effectiveness of bisoprolol and metoprolol succinate in patients with heart failure. *Int J Cardiol* 2015;**190**:4-6. doi: 10.1016/j.ijcard.2015.03.441
